# Dislocation of the Lower Jaw

**Published:** 1851-07

**Authors:** 


### ARTICLE XXX.

### *Dislocation of the Lower Jaw.*

An important addition has been made to practical surgery, in the introduction, by M. Nélaton, of a new, simple and efficacious process for the reduction of dislocation of the lower jaw.

According, then, to M. Nélaton, the obstacle to reduction, existing neither in the resistance offered to the condyle by the eminence of the transverse apophysis, nor in muscular action, it must be sought for, not in the articulation itself, but in neighboring parts.

"In front of the temporo-maxillary articulation," says M. Nélaton, "we find the temporo-zygomatic fossa, in which the coronoid process is lodged when the mouth is closed. Before and behind this excavation are placed two eminences, the posterior formed by the transverse root of the zygoma, the anterior by the articulation of the superior maxillary with the malar

* Drops of chloroform are widely different from minims, being extremely small.

bone. At the inferior part of the suture which results from the union of these two bones, there exists a tubercle sufficiently prominent, limited within by a notch formed by the smooth edge of the malar process of the superior maxillary bone, and often on the outer side by a little, elongated, almost oval fossette. This eminence, to which we may give the name of malar tubercle, is situated at about the distance of a centimetre from the coronoid process. In place of this tubercle we have sometimes met a plain surface, and even in certain subjects a notch more or less deep; but the presence of the tubercle is the rule. With regard to the coronoid process this latter presents great differences; very short in some, and elevated scarcely to the level of the condyle, it is found very much elongated in others; sometimes directed upwards, at other times obliquely outwards, so that its summit tends to meet the zygomatic arch; in some instances directed forwards, and distant from the condyle; in others directed backwards, so as to approach it." "These facts, well established," continues M. Nélaton, "let us examine the pathological condition. Having, as I have said, undertaken some experiments on the dead body, with a view to verify the prevailing doctrine on luxations of the lower jaw, I have ascertained: 1st, as M. Malgaigne observes, that if the condyle of the lower jaw is carried forwards without passing the point which the cavity of the capsule permits it to reach, the displacement disappears forcibly as soon as we approximate the dental arches, the eminence of the transverse apophysis presenting no obstacle to the return of the condyle. 2d, that if the anterior part of the capsule be cut or torn, so that the condyle can pass out of it and advance a few millimetres, we remark that the displacement is permanent, not, as is generally believed, because of the elevation of the transverse root, nor by reason of the contraction or tension of the muscles, but because the summit of the coronoid process comes to butt *(arc-bouter)* against the inferior and anterior angle of the malar bone, and is lodged in the little fossette which we have said exists often at this point. The contact of the summit of the coronoid process with the malar bone appears to us, then, to constitute an indispensable condition in the true dislocation; and for this the displacement need not be extreme; it suffices that the condyle be advanced from three to four millimetres. The external lateral ligament remains intact, the capsule is torn at its anterior part, and the inter-articular cartilage either accompanies the condyle in its displacement, or remains beneath the transverse root, according as the rupture is either above or below its anterior edge. . . . . It results from what precedes that it is not on the condyle that we must fix attention to find the cause which renders the dislocation permanent, but on the coronoid process and the malar bone, since it is in the contact of these two bones that almost all the difficulty of reduction resides."

To succeed, according to the process of M. Nélaton, it is necessary to act either by the interior of the mouth, taking a point of support behind the mastoid processes, or externally, by the operator taking a position behind the patient, and making pressure on the coronoid process, pushing it downwards and backwards, to disengage it from contact with the malar bone, at the same time that the patient opens the mouth. In ordinary cases, a light pressure is sufficient; but if more force be requisite, M. Nélaton advises the head to be supported by an assistant, or a band to be passed around it, in which the operator can engage his index and middle fingers, while the thumb must be brought to bear on the coronoid process.

The method of reduction which we have now described has been before the profession for more than a year. We have seen the success which has attended its use in the author's hands; and when we reflect on the great difficulties frequently presented to the surgeon in attempting to restore the lower jaw to the glenoid cavity, we cannot but feel that M. Nélaton will have conferred a signal benefit on humanity, should the rigid test of experience prove that his anticipations of the facility of reduction by the method he has devised, are as well founded as we are confident his views of the pathology of this luxation are correct. We shall anxiously await the verdict of the clinical surgeons of Dublin on the subject.—*Southern Med. and Surg. Journal.*

